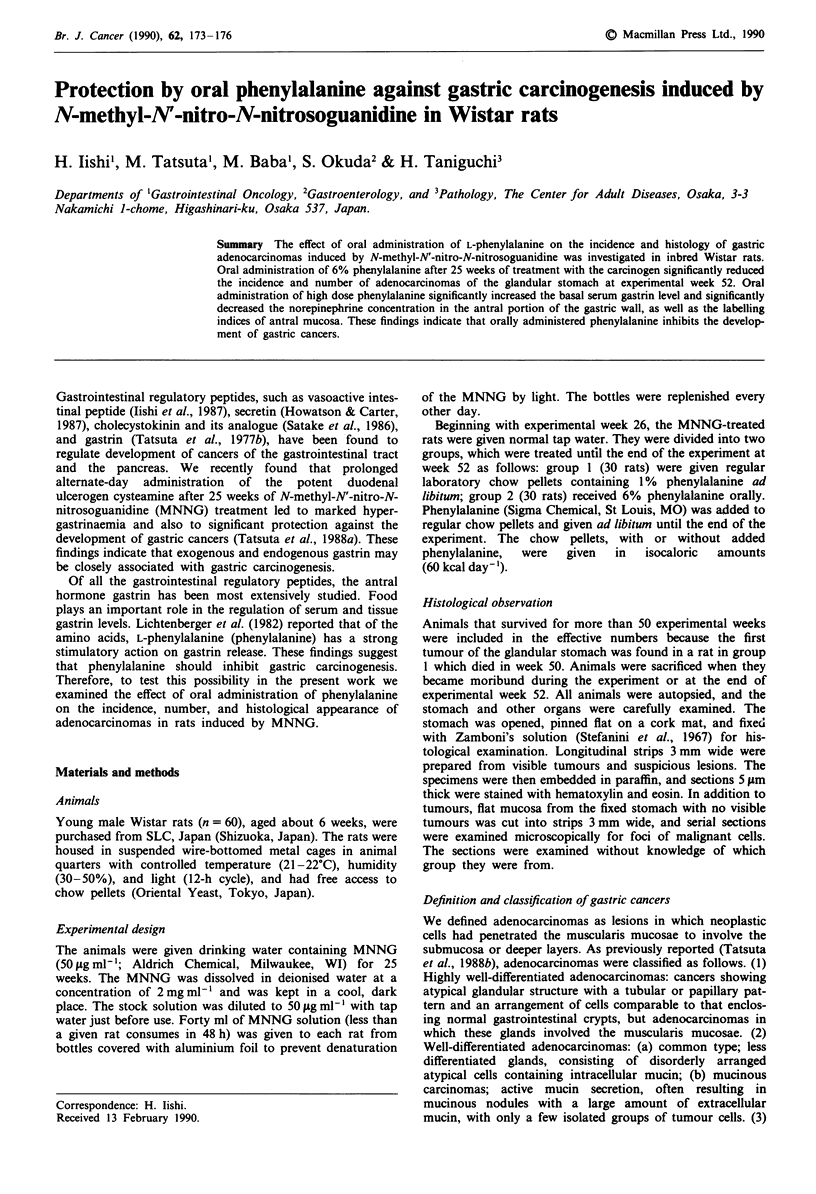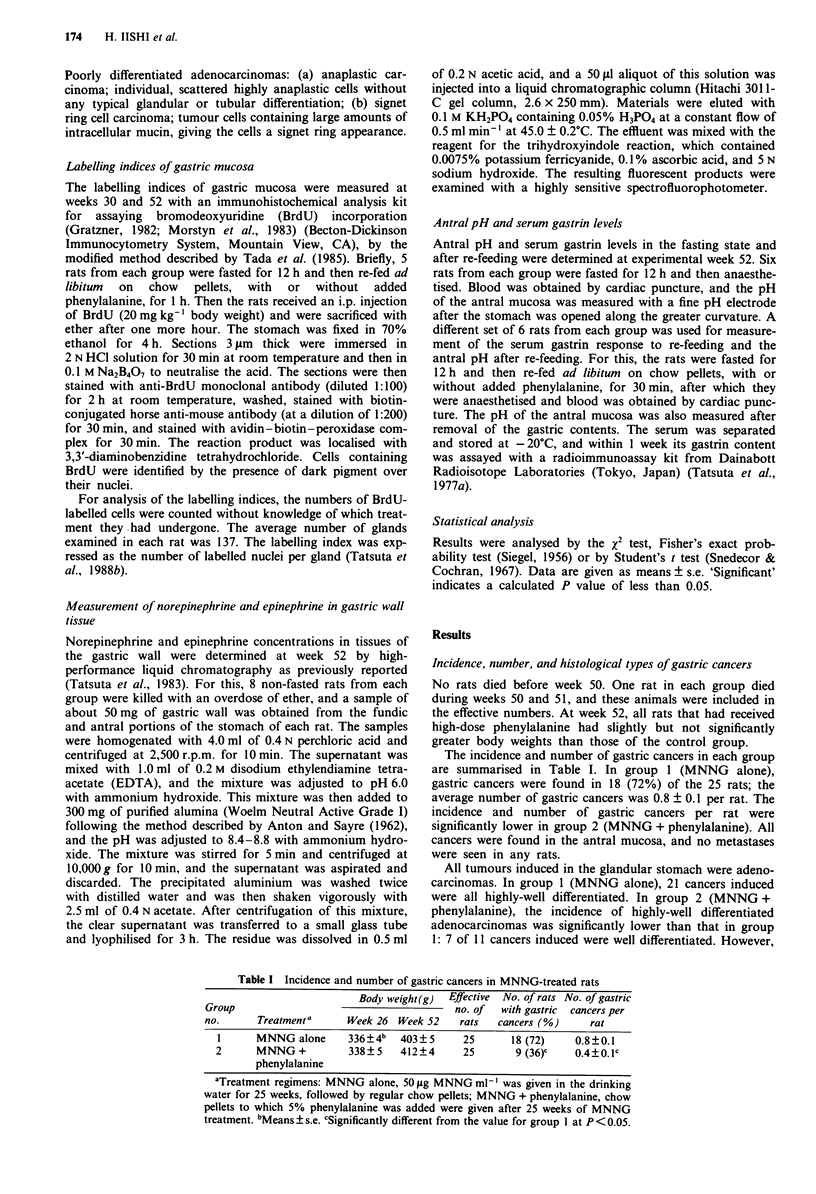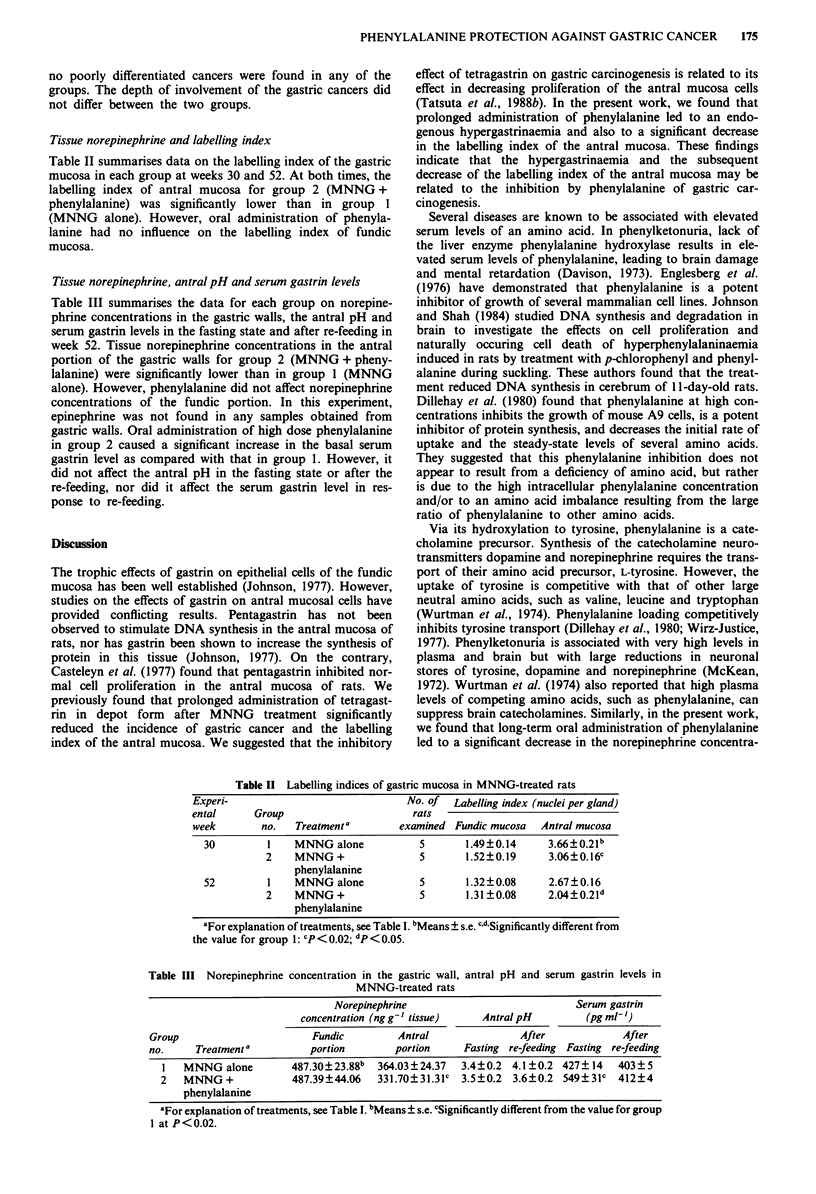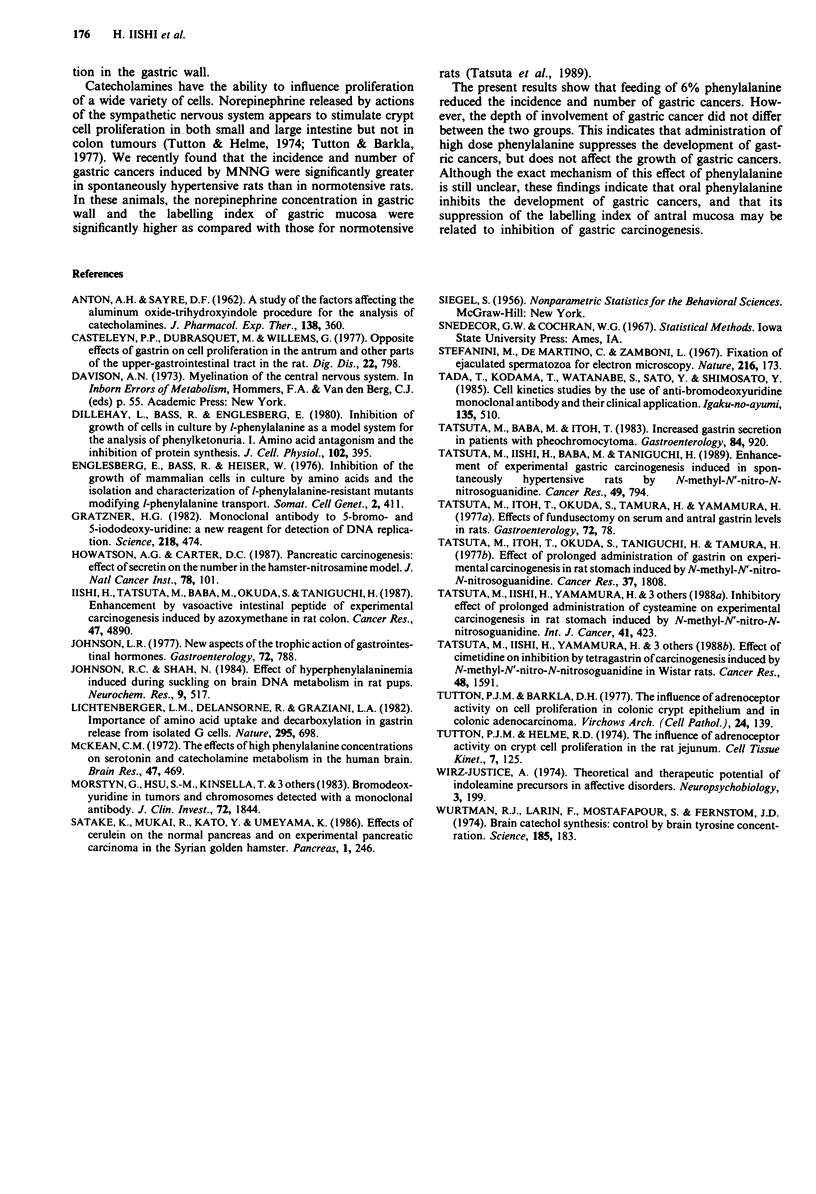# Protection by oral phenylalanine against gastric carcinogenesis induced by N-methyl-N'-nitro-N-nitrosoguanidine in Wistar rats.

**DOI:** 10.1038/bjc.1990.255

**Published:** 1990-08

**Authors:** H. Iishi, M. Tatsuta, M. Baba, S. Okuda, H. Taniguchi

**Affiliations:** Department of Gastrointestinal Oncology, Center for Adult Diseases, Osaka, Japan.

## Abstract

The effect of oral administration of L-phenylalanine on the incidence and histology of gastric adenocarcinomas induced by N-methyl-N'-nitro-N-nitrosoguanidine was investigated in inbred Wistar rats. Oral administration of 6% phenylalanine after 25 weeks of treatment with the carcinogen significantly reduced the incidence and number of adenocarcinomas of the glandular stomach at experimental week 52. Oral administration of high dose phenylalanine significantly increased the basal serum gastrin level and significantly decreased the norepinephrine concentration in the antral portion of the gastric wall, as well as the labelling indices of antral mucosa. These findings indicate that orally administered phenylalanine inhibits the development of gastric cancers.


Br. J. Cancer (1990), 62, 173-176 © Macmillan Press Ltd., 1990

### Protection by oral phenylalanine against gastric carcinogenesis induced by *N*-methyl-*N'*-nitro-*N*-nitrosoguanidine in Wistar rats

H. Iishi[1], M. Tatsuta[1], M. Baba[1], S. Okuda[2] & H. Taniguchi[3]

*Departments of [1]Gastrointestinal Oncology, [2]Gastroenterology, and [3]Pathology, The Center for Adult Diseases, Osaka, 3-3 Nakamichi 1-chome, Higashinari-ku, Osaka 537, Japan.*

**Summary** The effect of oral administration of L-phenylalanine on the incidence and histology of gastric adenocarcinomas induced by *N*-methyl-*N'*-nitro-*N*-nitrosoguanidine was investigated in inbred Wistar rats. Oral administration of 6% phenylalanine after 25 weeks of treatment with the carcinogen significantly reduced the incidence and number of adenocarcinomas of the glandular stomach at experimental week 52. Oral administration of high dose phenylalanine significantly increased the basal serum gastrin level and significantly decreased the norepinephrine concentration in the antral portion of the gastric wall, as well as the labelling indices of antral mucosa. These findings indicate that orally administered phenylalanine inhibits the development of gastric cancers.

Gastrointestinal regulatory peptides, such as vasoactive intestinal peptide (Iishi *et al.*, 1987), secretin (Howatson & Carter, 1987), cholecystokinin and its analogue (Satake *et al.*, 1986), and gastrin (Tatsuta *et al.*, 1977*b*), have been found to regulate development of cancers of the gastrointestinal tract and the pancreas. We recently found that prolonged alternate-day administration of the potent duodenal ulcerogen cysteamine after 25 weeks of *N*-methyl-*N'*-nitro-*N*-nitrosoguanidine (MNNG) treatment led to marked hypergastrinaemia and also to significant protection against the development of gastric cancers (Tatsuta *et al.*, 1988*a*). These findings indicate that exogenous and endogenous gastrin may be closely associated with gastric carcinogenesis.

Of all the gastrointestinal regulatory peptides, the antral hormone gastrin has been most extensively studied. Food plays an important role in the regulation of serum and tissue gastrin levels. Lichtenberger *et al.* (1982) reported that of the amino acids, L-phenylalanine (phenylalanine) has a strong stimulatory action on gastrin release. These findings suggest that phenylalanine should inhibit gastric carcinogenesis. Therefore, to test this possibility in the present work we examined the effect of oral administration of phenylalanine on the incidence, number, and histological appearance of adenocarcinomas in rats induced by MNNG.

### Materials and methods

### *Animals*

Young male Wistar rats ($n = 60$), aged about 6 weeks, were purchased from SLC, Japan (Shizuoka, Japan). The rats were housed in suspended wire-bottomed metal cages in animal quarters with controlled temperature (21-22°C), humidity (30-50%), and light (12-h cycle), and had free access to chow pellets (Oriental Yeast, Tokyo, Japan).

### *Experimental design*

The animals were given drinking water containing MNNG (50 $\mu g\ ml^{-1}$; Aldrich Chemical, Milwaukee, WI) for 25 weeks. The MNNG was dissolved in deionised water at a concentration of 2 $mg\ ml^{-1}$ and was kept in a cool, dark place. The stock solution was diluted to 50 $\mu g\ ml^{-1}$ with tap water just before use. Forty ml of MNNG solution (less than a given rat consumes in 48 h) was given to each rat from bottles covered with aluminium foil to prevent denaturation of the MNNG by light. The bottles were replenished every other day.

Beginning with experimental week 26, the MNNG-treated rats were given normal tap water. They were divided into two groups, which were treated until the end of the experiment at week 52 as follows: group 1 (30 rats) were given regular laboratory chow pellets containing 1% phenylalanine *ad libitum*; group 2 (30 rats) received 6% phenylalanine orally. Phenylalanine (Sigma Chemical, St Louis, MO) was added to regular chow pellets and given *ad libitum* until the end of the experiment. The chow pellets, with or without added phenylalanine, were given in isocaloric amounts (60 $kcal\ day^{-1}$).

### *Histological observation*

Animals that survived for more than 50 experimental weeks were included in the effective numbers because the first tumour of the glandular stomach was found in a rat in group 1 which died in week 50. Animals were sacrificed when they became moribund during the experiment or at the end of experimental week 52. All animals were autopsied, and the stomach and other organs were carefully examined. The stomach was opened, pinned flat on a cork mat, and fixed with Zamboni's solution (Stefanini *et al.*, 1967) for histological examination. Longitudinal strips 3 mm wide were prepared from visible tumours and suspicious lesions. The specimens were then embedded in paraffin, and sections 5 $\mu m$ thick were stained with hematoxylin and eosin. In addition to tumours, flat mucosa from the fixed stomach with no visible tumours was cut into strips 3 mm wide, and serial sections were examined microscopically for foci of malignant cells. The sections were examined without knowledge of which group they were from.

### *Definition and classification of gastric cancers*

We defined adenocarcinomas as lesions in which neoplastic cells had penetrated the muscularis mucosae to involve the submucosa or deeper layers. As previously reported (Tatsuta *et al.*, 1988*b*), adenocarcinomas were classified as follows. (1) Highly well-differentiated adenocarcinomas: cancers showing atypical glandular structure with a tubular or papillary pattern and an arrangement of cells comparable to that enclosing normal gastrointestinal crypts, but adenocarcinomas in which these glands involved the muscularis mucosae. (2) Well-differentiated adenocarcinomas: (a) common type; less differentiated glands, consisting of disorderly arranged atypical cells containing intracellular mucin; (b) mucinous carcinomas; active mucin secretion, often resulting in mucinous nodules with a large amount of extracellular mucin, with only a few isolated groups of tumour cells. (3)

Correspondence: H. Iishi.

Received 13 February 1990.

Poorly differentiated adenocarcinomas: (a) anaplastic carcinoma; individual, scattered highly anaplastic cells without any typical glandular or tubular differentiation; (b) signet ring cell carcinoma; tumour cells containing large amounts of intracellular mucin, giving the cells a signet ring appearance.

*Labelling indices of gastric mucosa*

The labelling indices of gastric mucosa were measured at weeks 30 and 52 with an immunohistochemical analysis kit for assaying bromodeoxyuridine (BrdU) incorporation (Gratzner, 1982; Morstyn *et al.*, 1983) (Becton-Dickinson Immunocytometry System, Mountain View, CA), by the modified method described by Tada *et al.* (1985). Briefly, 5 rats from each group were fasted for 12 h and then re-fed *ad libitum* on chow pellets, with or without added phenylalanine, for 1 h. Then the rats received an i.p. injection of BrdU (20 mg $kg^{-1}$ body weight) and were sacrificed with ether after one more hour. The stomach was fixed in 70% ethanol for 4 h. Sections 3 μm thick were immersed in 2 N HCl solution for 30 min at room temperature and then in 0.1 M $Na_2B_4O_7$ to neutralise the acid. The sections were then stained with anti-BrdU monoclonal antibody (diluted 1:100) for 2 h at room temperature, washed, stained with biotin-conjugated horse anti-mouse antibody (at a dilution of 1:200) for 30 min, and stained with avidin–biotin–peroxidase complex for 30 min. The reaction product was localised with 3,3′-diaminobenzidine tetrahydrochloride. Cells containing BrdU were identified by the presence of dark pigment over their nuclei.

For analysis of the labelling indices, the numbers of BrdU-labelled cells were counted without knowledge of which treatment they had undergone. The average number of glands examined in each rat was 137. The labelling index was expressed as the number of labelled nuclei per gland (Tatsuta *et al.*, 1988*b*).

*Measurement of norepinephrine and epinephrine in gastric wall tissue*

Norepinephrine and epinephrine concentrations in tissues of the gastric wall were determined at week 52 by high-performance liquid chromatography as previously reported (Tatsuta *et al.*, 1983). For this, 8 non-fasted rats from each group were killed with an overdose of ether, and a sample of about 50 mg of gastric wall was obtained from the fundic and antral portions of the stomach of each rat. The samples were homogenated with 4.0 ml of 0.4 N perchloric acid and centrifuged at 2,500 r.p.m. for 10 min. The supernatant was mixed with 1.0 ml of 0.2 M disodium ethylendiamine tetra-acetate (EDTA), and the mixture was adjusted to pH 6.0 with ammonium hydroxide. This mixture was then added to 300 mg of purified alumina (Woelm Neutral Active Grade I) following the method described by Anton and Sayre (1962), and the pH was adjusted to 8.4–8.8 with ammonium hydroxide. The mixture was stirred for 5 min and centrifuged at 10,000 *g* for 10 min, and the supernatant was aspirated and discarded. The precipitated aluminium was washed twice with distilled water and was then shaken vigorously with 2.5 ml of 0.4 N acetate. After centrifugation of this mixture, the clear supernatant was transferred to a small glass tube and lyophilised for 3 h. The residue was dissolved in 0.5 ml of 0.2 N acetic acid, and a 50 μl aliquot of this solution was injected into a liquid chromatographic column (Hitachi 3011-C gel column, 2.6 × 250 mm). Materials were eluted with 0.1 M $KH_2PO_4$ containing 0.05% $H_3PO_4$ at a constant flow of 0.5 ml $min^{-1}$ at 45.0 ± 0.2°C. The effluent was mixed with the reagent for the trihydroxyindole reaction, which contained 0.0075% potassium ferricyanide, 0.1% ascorbic acid, and 5 N sodium hydroxide. The resulting fluorescent products were examined with a highly sensitive spectrofluorophotometer.

*Antral pH and serum gastrin levels*

Antral pH and serum gastrin levels in the fasting state and after re-feeding were determined at experimental week 52. Six rats from each group were fasted for 12 h and then anaesthetised. Blood was obtained by cardiac puncture, and the pH of the antral mucosa was measured with a fine pH electrode after the stomach was opened along the greater curvature. A different set of 6 rats from each group was used for measurement of the serum gastrin response to re-feeding and the antral pH after re-feeding. For this, the rats were fasted for 12 h and then re-fed *ad libitum* on chow pellets, with or without added phenylalanine, for 30 min, after which they were anaesthetised and blood was obtained by cardiac puncture. The pH of the antral mucosa was also measured after removal of the gastric contents. The serum was separated and stored at −20°C, and within 1 week its gastrin content was assayed with a radioimmunoassay kit from Dainabott Radioisotope Laboratories (Tokyo, Japan) (Tatsuta *et al.*, 1977*a*).

*Statistical analysis*

Results were analysed by the $\chi^2$ test, Fisher's exact probability test (Siegel, 1956) or by Student's *t* test (Snedecor & Cochran, 1967). Data are given as means ± s.e. 'Significant' indicates a calculated $P$ value of less than 0.05.

### Results

*Incidence, number, and histological types of gastric cancers*

No rats died before week 50. One rat in each group died during weeks 50 and 51, and these animals were included in the effective numbers. At week 52, all rats that had received high-dose phenylalanine had slightly but not significantly greater body weights than those of the control group.

The incidence and number of gastric cancers in each group are summarised in Table I. In group 1 (MNNG alone), gastric cancers were found in 18 (72%) of the 25 rats; the average number of gastric cancers was 0.8 ± 0.1 per rat. The incidence and number of gastric cancers per rat were significantly lower in group 2 (MNNG + phenylalanine). All cancers were found in the antral mucosa, and no metastases were seen in any rats.

All tumours induced in the glandular stomach were adenocarcinomas. In group 1 (MNNG alone), 21 cancers induced were all highly-well differentiated. In group 2 (MNNG + phenylalanine), the incidence of highly-well differentiated adenocarcinomas was significantly lower than that in group 1: 7 of 11 cancers induced were well differentiated. However,

**Table I** Incidence and number of gastric cancers in MNNG-treated rats

| Group no. | Treatment[a] | Body weight (g) Week 26 | Body weight (g) Week 52 | Effective no. of rats | No. of rats with gastric cancers (%) | No. of gastric cancers per rat |
|---|---|---|---|---|---|---|
| 1 | MNNG alone | 336±4[b] | 403±5 | 25 | 18 (72) | 0.8±0.1 |
| 2 | MNNG + phenylalanine | 338±5 | 412±4 | 25 | 9 (36)[c] | 0.4±0.1[c] |

[a]Treatment regimens: MNNG alone, 50 μg MNNG $ml^{-1}$ was given in the drinking water for 25 weeks, followed by regular chow pellets; MNNG + phenylalanine, chow pellets to which 5% phenylalanine was added were given after 25 weeks of MNNG treatment. [b]Means ± s.e. [c]Significantly different from the value for group 1 at $P < 0.05$.

no poorly differentiated cancers were found in any of the groups. The depth of involvement of the gastric cancers did not differ between the two groups.

*Tissue norepinephrine and labelling index*

Table II summarises data on the labelling index of the gastric mucosa in each group at weeks 30 and 52. At both times, the labelling index of antral mucosa for group 2 (MNNG + phenylalanine) was significantly lower than in group 1 (MNNG alone). However, oral administration of phenylalanine had no influence on the labelling index of fundic mucosa.

*Tissue norepinephrine, antral pH and serum gastrin levels*

Table III summarises the data for each group on norepinephrine concentrations in the gastric walls, the antral pH and serum gastrin levels in the fasting state and after re-feeding in week 52. Tissue norepinephrine concentrations in the antral portion of the gastric walls for group 2 (MNNG + phenylalanine) were significantly lower than in group 1 (MNNG alone). However, phenylalanine did not affect norepinephrine concentrations of the fundic portion. In this experiment, epinephrine was not found in any samples obtained from gastric walls. Oral administration of high dose phenylalanine in group 2 caused a significant increase in the basal serum gastrin level as compared with that in group 1. However, it did not affect the antral pH in the fasting state or after the re-feeding, nor did it affect the serum gastrin level in response to re-feeding.

### Discussion

The trophic effects of gastrin on epithelial cells of the fundic mucosa has been well established (Johnson, 1977). However, studies on the effects of gastrin on antral mucosal cells have provided conflicting results. Pentagastrin has not been observed to stimulate DNA synthesis in the antral mucosa of rats, nor has gastrin been shown to increase the synthesis of protein in this tissue (Johnson, 1977). On the contrary, Casteleyn *et al.* (1977) found that pentagastrin inhibited normal cell proliferation in the antral mucosa of rats. We previously found that prolonged administration of tetragastrin in depot form after MNNG treatment significantly reduced the incidence of gastric cancer and the labelling index of the antral mucosa. We suggested that the inhibitory effect of tetragastrin on gastric carcinogenesis is related to its effect in decreasing proliferation of the antral mucosa cells (Tatsuta *et al.*, 1988*b*). In the present work, we found that prolonged administration of phenylalanine led to an endogenous hypergastrinaemia and also to a significant decrease in the labelling index of the antral mucosa. These findings indicate that the hypergastrinaemia and the subsequent decrease of the labelling index of the antral mucosa may be related to the inhibition by phenylalanine of gastric carcinogenesis.

Several diseases are known to be associated with elevated serum levels of an amino acid. In phenylketonuria, lack of the liver enzyme phenylalanine hydroxylase results in elevated serum levels of phenylalanine, leading to brain damage and mental retardation (Davison, 1973). Englesberg *et al.* (1976) have demonstrated that phenylalanine is a potent inhibitor of growth of several mammalian cell lines. Johnson and Shah (1984) studied DNA synthesis and degradation in brain to investigate the effects on cell proliferation and naturally occuring cell death of hyperphenylalaninaemia induced in rats by treatment with *p*-chlorophenyl and phenylalanine during suckling. These authors found that the treatment reduced DNA synthesis in cerebrum of 11-day-old rats. Dillehay *et al.* (1980) found that phenylalanine at high concentrations inhibits the growth of mouse A9 cells, is a potent inhibitor of protein synthesis, and decreases the initial rate of uptake and the steady-state levels of several amino acids. They suggested that this phenylalanine inhibition does not appear to result from a deficiency of amino acid, but rather is due to the high intracellular phenylalanine concentration and/or to an amino acid imbalance resulting from the large ratio of phenylalanine to other amino acids.

Via its hydroxylation to tyrosine, phenylalanine is a catecholamine precursor. Synthesis of the catecholamine neurotransmitters dopamine and norepinephrine requires the transport of their amino acid precursor, L-tyrosine. However, the uptake of tyrosine is competitive with that of other large neutral amino acids, such as valine, leucine and tryptophan (Wurtman *et al.*, 1974). Phenylalanine loading competitively inhibits tyrosine transport (Dillehay *et al.*, 1980; Wirz-Justice, 1977). Phenylketonuria is associated with very high levels in plasma and brain but with large reductions in neuronal stores of tyrosine, dopamine and norepinephrine (McKean, 1972). Wurtman *et al.* (1974) also reported that high plasma levels of competing amino acids, such as phenylalanine, can suppress brain catecholamines. Similarly, in the present work, we found that long-term oral administration of phenylalanine led to a significant decrease in the norepinephrine concentra-

**Table II** Labelling indices of gastric mucosa in MNNG-treated rats

| *Experimental week* | *Group no.* | *Treatment*[a] | *No. of rats examined* | *Labelling index (nuclei per gland)* Fundic mucosa | Antral mucosa |
|---|---|---|---|---|---|
| 30 | 1 | MNNG alone | 5 | 1.49±0.14 | 3.66±0.21[b] |
| | 2 | MNNG + phenylalanine | 5 | 1.52±0.19 | 3.06±0.16[c] |
| 52 | 1 | MNNG alone | 5 | 1.32±0.08 | 2.67±0.16 |
| | 2 | MNNG + phenylalanine | 5 | 1.31±0.08 | 2.04±0.21[d] |

[a]For explanation of treatments, see Table I. [b]Means ± s.e. [c,d]Significantly different from the value for group 1: [c]$P<0.02$; [d]$P<0.05$.

**Table III** Norepinephrine concentration in the gastric wall, antral pH and serum gastrin levels in MNNG-treated rats

| *Group no.* | *Treatment*[a] | *Norepinephrine concentration (ng g$^{-1}$ tissue)* Fundic portion | Antral portion | *Antral pH* Fasting | After re-feeding | *Serum gastrin (pg ml$^{-1}$)* Fasting | After re-feeding |
|---|---|---|---|---|---|---|---|
| 1 | MNNG alone | 487.30±23.88[b] | 364.03±24.37 | 3.4±0.2 | 4.1±0.2 | 427±14 | 403±5 |
| 2 | MNNG + phenylalanine | 487.39±44.06 | 331.70±31.31[c] | 3.5±0.2 | 3.6±0.2 | 549±31[c] | 412±4 |

[a]For explanation of treatments, see Table I. [b]Means ± s.e. [c]Significantly different from the value for group 1 at $P<0.02$.

tion in the gastric wall.

Catecholamines have the ability to influence proliferation of a wide variety of cells. Norepinephrine released by actions of the sympathetic nervous system appears to stimulate crypt cell proliferation in both small and large intestine but not in colon tumours (Tutton & Helme, 1974; Tutton & Barkla, 1977). We recently found that the incidence and number of gastric cancers induced by MNNG were significantly greater in spontaneously hypertensive rats than in normotensive rats. In these animals, the norepinephrine concentration in gastric wall and the labelling index of gastric mucosa were significantly higher as compared with those for normotensive rats (Tatsuta et al., 1989).

The present results show that feeding of 6% phenylalanine reduced the incidence and number of gastric cancers. However, the depth of involvement of gastric cancer did not differ between the two groups. This indicates that administration of high dose phenylalanine suppresses the development of gastric cancers, but does not affect the growth of gastric cancers. Although the exact mechanism of this effect of phenylalanine is still unclear, these findings indicate that oral phenylalanine inhibits the development of gastric cancers, and that its suppression of the labelling index of antral mucosa may be related to inhibition of gastric carcinogenesis.

### References

ANTON, A.H. & SAYRE, D.F. (1962). A study of the factors affecting the aluminum oxide-trihydroxyindole procedure for the analysis of catecholamines. *J. Pharmacol. Exp. Ther.*, **138**, 360.

CASTELEYN, P.P., DUBRASQUET, M. & WILLEMS, G. (1977). Opposite effects of gastrin on cell proliferation in the antrum and other parts of the upper-gastrointestinal tract in the rat. *Dig. Dis.*, **22**, 798.

DAVISON, A.N. (1973). Myelination of the central nervous system. In *Inborn Errors of Metabolism*, Hommers, F.A. & Van den Berg, C.J. (eds) p. 55. Academic Press: New York.

DILLEHAY, L., BASS, R. & ENGLESBERG, E. (1980). Inhibition of growth of cells in culture by *l*-phenylalanine as a model system for the analysis of phenylketonuria. I. Amino acid antagonism and the inhibition of protein synthesis. *J. Cell. Physiol.*, **102**, 395.

ENGLESBERG, E., BASS, R. & HEISER, W. (1976). Inhibition of the growth of mammalian cells in culture by amino acids and the isolation and characterization of *l*-phenylalanine-resistant mutants modifying *l*-phenylalanine transport. *Somat. Cell Genet.*, **2**, 411.

GRATZNER, H.G. (1982). Monoclonal antibody to 5-bromo- and 5-iododeoxy-uridine: a new reagent for detection of DNA replication. *Science*, **218**, 474.

HOWATSON, A.G. & CARTER, D.C. (1987). Pancreatic carcinogenesis: effect of secretin on the number in the hamster-nitrosamine model. *J. Natl Cancer Inst.*, **78**, 101.

IISHI, H., TATSUTA, M., BABA, M., OKUDA, S. & TANIGUCHI, H. (1987). Enhancement by vasoactive intestinal peptide of experimental carcinogenesis induced by azoxymethane in rat colon. *Cancer Res.*, **47**, 4890.

JOHNSON, L.R. (1977). New aspects of the trophic action of gastrointestinal hormones. *Gastroenterology*, **72**, 788.

JOHNSON, R.C. & SHAH, N. (1984). Effect of hyperphenylalaninemia induced during suckling on brain DNA metabolism in rat pups. *Neurochem. Res.*, **9**, 517.

LICHTENBERGER, L.M., DELANSORNE, R. & GRAZIANI, L.A. (1982). Importance of amino acid uptake and decarboxylation in gastrin release from isolated G cells. *Nature*, **295**, 698.

McKEAN, C.M. (1972). The effects of high phenylalanine concentrations on serotonin and catecholamine metabolism in the human brain. *Brain Res.*, **47**, 469.

MORSTYN, G., HSU, S.-M., KINSELLA, T. & 3 others (1983). Bromodeoxyuridine in tumors and chromosomes detected with a monoclonal antibody. *J. Clin. Invest.*, **72**, 1844.

SATAKE, K., MUKAI, R., KATO, Y. & UMEYAMA, K. (1986). Effects of cerulein on the normal pancreas and on experimental pancreatic carcinoma in the Syrian golden hamster. *Pancreas*, **1**, 246.

SIEGEL, S. (1956). *Nonparametric Statistics for the Behavioral Sciences*. McGraw-Hill: New York.

SNEDECOR, G.W. & COCHRAN, W.G. (1967). *Statistical Methods*. Iowa State University Press: Ames, IA.

STEFANINI, M., DE MARTINO, C. & ZAMBONI, L. (1967). Fixation of ejaculated spermatozoa for electron microscopy. *Nature*, **216**, 173.

TADA, T., KODAMA, T., WATANABE, S., SATO, Y. & SHIMOSATO, Y. (1985). Cell kinetics studies by the use of anti-bromodeoxyuridine monoclonal antibody and their clinical application. *Igaku-no-ayumi*, **135**, 510.

TATSUTA, M., BABA, M. & ITOH, T. (1983). Increased gastrin secretion in patients with pheochromocytoma. *Gastroenterology*, **84**, 920.

TATSUTA, M., IISHI, H., BABA, M. & TANIGUCHI, H. (1989). Enhancement of experimental gastric carcinogenesis induced in spontaneously hypertensive rats by *N*-methyl-*N'*-nitro-*N*-nitrosoguanidine. *Cancer Res.*, **49**, 794.

TATSUTA, M., ITOH, T., OKUDA, S., TAMURA, H. & YAMAMURA, H. (1977*a*). Effects of fundusectomy on serum and antral gastrin levels in rats. *Gastroenterology*, **72**, 78.

TATSUTA, M., ITOH, T., OKUDA, S., TANIGUCHI, H. & TAMURA, H. (1977*b*). Effect of prolonged administration of gastrin on experimental carcinogenesis in rat stomach induced by *N*-methyl-*N'*-nitro-*N*-nitrosoguanidine. *Cancer Res.*, **37**, 1808.

TATSUTA, M., IISHI, H., YAMAMURA, H. & 3 others (1988*a*). Inhibitory effect of prolonged administration of cysteamine on experimental carcinogenesis in rat stomach induced by *N*-methyl-*N'*-nitro-*N*-nitrosoguanidine. *Int. J. Cancer*, **41**, 423.

TATSUTA, M., IISHI, H., YAMAMURA, H. & 3 others (1988*b*). Effect of cimetidine on inhibition by tetragastrin of carcinogenesis induced by *N*-methyl-*N'*-nitro-*N*-nitrosoguanidine in Wistar rats. *Cancer Res.*, **48**, 1591.

TUTTON, P.J.M. & BARKLA, D.H. (1977). The influence of adrenoceptor activity on cell proliferation in colonic crypt epithelium and in colonic adenocarcinoma. *Virchows Arch. (Cell Pathol.)*, **24**, 139.

TUTTON, P.J.M. & HELME, R.D. (1974). The influence of adrenoceptor activity on crypt cell proliferation in the rat jejunum. *Cell Tissue Kinet.*, **7**, 125.

WIRZ-JUSTICE, A. (1974). Theoretical and therapeutic potential of indoleamine precursors in affective disorders. *Neuropsychobiology*, **3**, 199.

WURTMAN, R.J., LARIN, F., MOSTAFAPOUR, S. & FERNSTOM, J.D. (1974). Brain catechol synthesis: control by brain tyrosine concentration. *Science*, **185**, 183.